# Supplementary material for: A role for spindles in the onset of rapid eye movement sleep
Source: Nat Commun. 2020 Oct 16;11:5247. doi: 10.1038/s41467-020-19076-2 (PMC7567828; doi:10.1038/s41467-020-19076-2)
Supplement: Supplementary file 1 — Supplementary Information [file 41467_2020_19076_MOESM1_ESM.pdf]

Supplementary information

A role for spindles in the onset of rapid eye movement sleep

Mojtaba Bandarabadi[1,2,4,†], Carolina Gutierrez Herrera[1,3,†], Thomas C. Gent[1,3,†], Claudio Bassetti[1,2], Kaspar Schindler[1,2], and Antoine R. Adamantidis[1,2,3,*]

[1] Zentrum für Experimentelle Neurologie, Department of Neurology, Inselspital University Hospital Bern, Bern, Switzerland.
[2] Sleep-Wake-Epilepsy Center, Department of Neurology, Inselspital University Hospital Bern, Bern, Switzerland.
[3] Department of Biomedical Research, University of Bern, Bern, Switzerland.
[4] Present address: Department of Biomedical Sciences, University of Lausanne, Lausanne, Switzerland.
[†] These authors contributed equally to this work.

[*] *Correspondence to:*
Antoine R. Adamantidis, PhD.
Center for Experimental Neurology
Department of Neurology
Inselspital University Hospital Bern
Freiburgstrasse 18
3010 Bern, Switzerland
Tel: +41 (0) 31 632 55 93
antoine.adamantidis@dbmr.unibe.ch

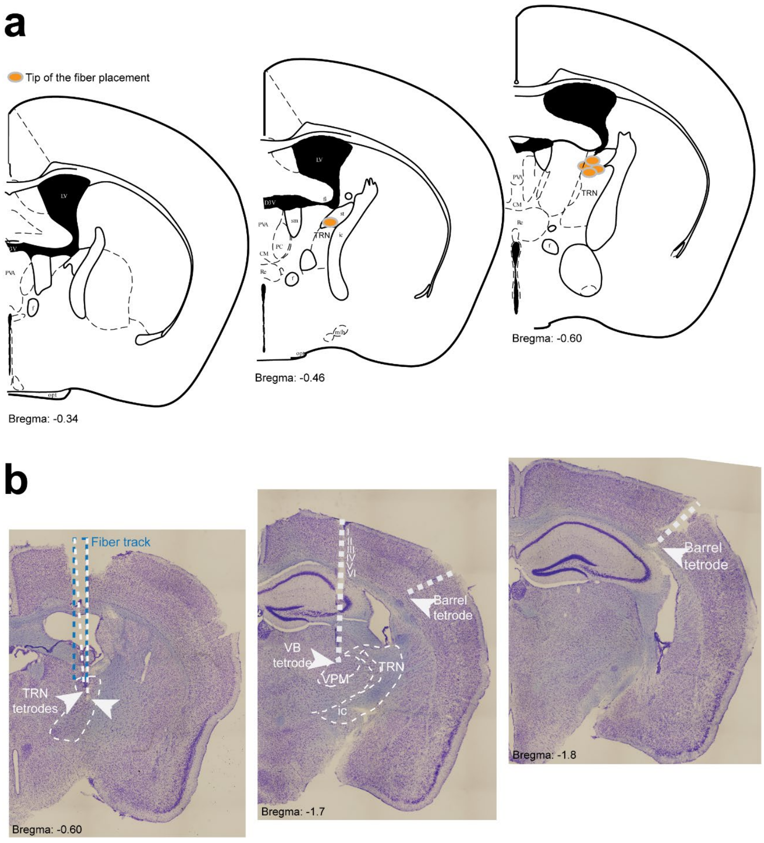

Supplementary Fig. 1. Placement of tetrodes and optic fiber for the TRN-VB-BARR recordings. (a) Schematic illustration of fiber optic placement for optogenetic experiments. The orange circles indicate tip of the fiber optic placement in the TRN. (b) Sample coronal sections from tetrodes implanted in the TRN, VB, and BARR, as well as the optic fiber in the TRN.

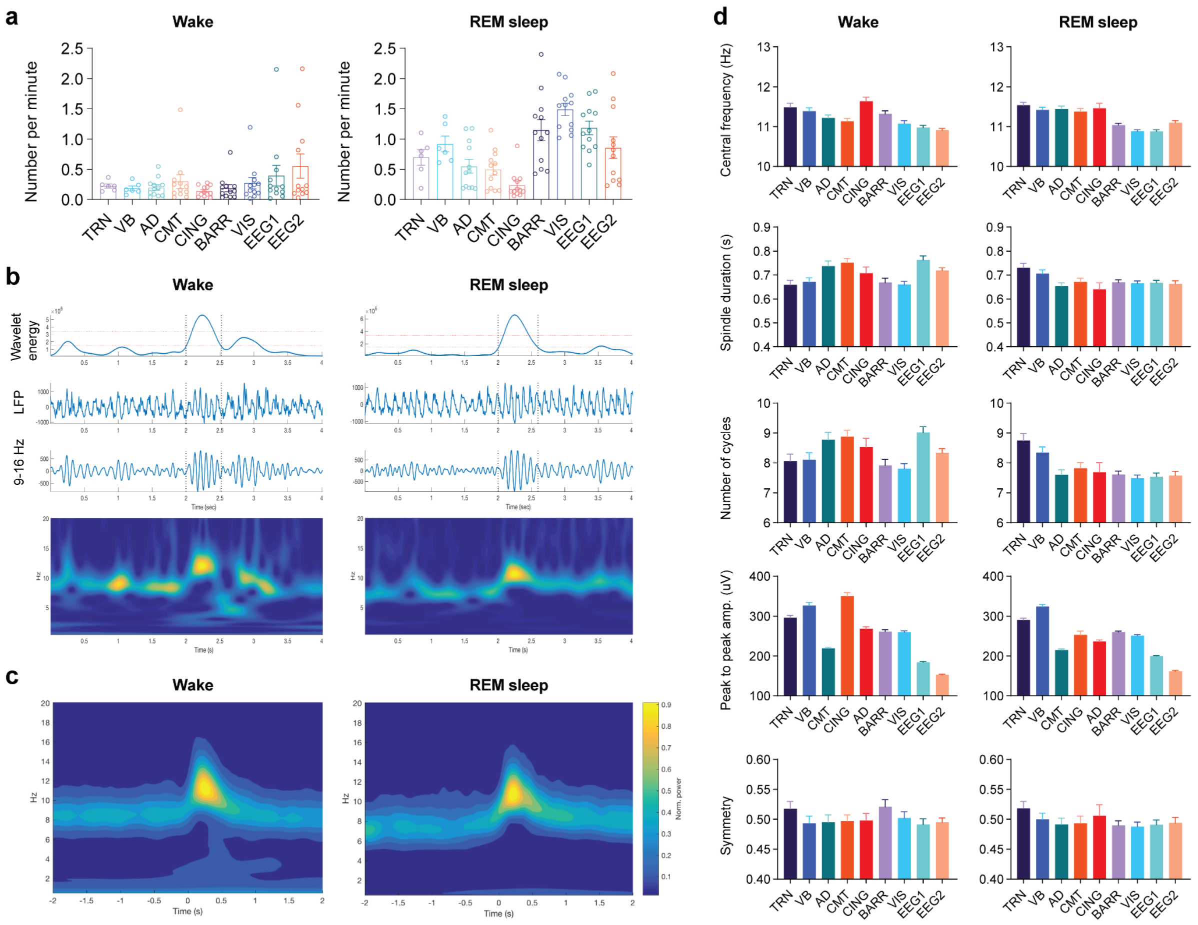

Supplementary Fig. 2. Spindle-like events during wakefulness and REM sleep. (a) Rate of the detected spindle-like events during wakefulness and REM sleep for each recording site (wake: TRN: 0.22 ± 0.03, VB: 0.19 ± 0.04, AD: 0.20 ± 0.04, CMT: 0.29 ± 0.11, CING: 0.14 ± 0.03, BARR: 0.19 ± 0.06, VIS: 0.27 ± 0.09, EEG1: 0.39 ± 0.17, EEG2: 0.55 ± 0.19; REM sleep: TRN: 0.69 ± 0.13, VB: 0.92 ± 0.13, AD: 0.55 ± 0.11, CMT: 0.49 ± 0.09, CING: 0.24 ± 0.07, BARR: 1.15 ± 0.17, VIS: 1.49 ± 0.10, EEG1: 1.18 ± 0.11, EEG2: 0.86 ± 0.18; n = 6 animals for TRN/VB and n = 12 for other sites). (b) Representative LFP, wavelet energy, filtered signal in the spindle range, and spectrogram of detected events during wakefulness and REM sleep. (c) Average spectrograms of detected events during wake and REM sleep. (d) Central frequency, duration, number of cycles, peak-to-peak amplitude, and the symmetric measures for the detected spindle-like events during wakefulness and REM sleep for each site (n = 6 animals for TRN/VB and n = 12 for other sites). Error bars indicate mean ± S.E.M.

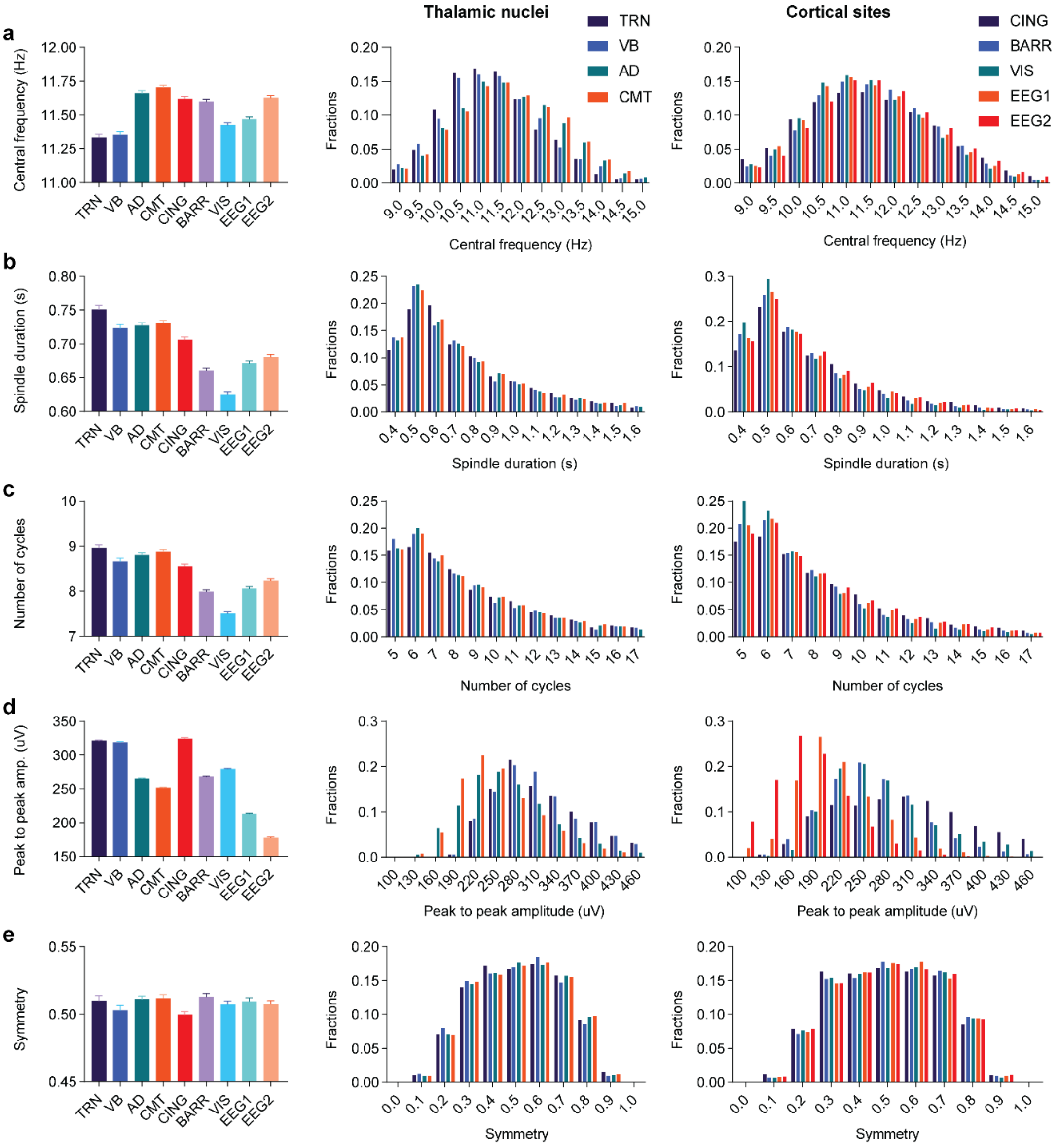

Supplementary Fig. 3. Characterization of regionally detected sleep spindles. (a) Central frequency of spindles for each site. Histograms represent distribution of central frequency across different frequency bins, separated for thalamic and cortical recordings. The central frequency of spindles is generally homogenous, from 11.3 ± 0.02 Hz for TRN to 11.7 ± 0.02 Hz for CMT, with no distinguishable clusters of slow and fast spindles in mice. (b-e) Same as (a), but for spindle duration, number of cycles, peak-to-peak amplitude, and the symmetric measure. The average spindle duration varied from 625 ± 3 ms (VIS) to 750 ± 6 ms (TRN) and showed a unimodal distribution, with maximal fractions around 500 ms. The average number of cycles is 8.4 ± 0.16 for cortical and thalamic sites, with maximal fractions having 6 cycles. The peak-to-peak amplitude is highest for the CING (324 ± 2 μV) and TRN (321 ± 2 μV) spindles, while lowest for the cortical EEG (178 ± 1 μV). Spindle waveforms are highly symmetric in all the recorded sites (5457 ± 73 spindles, 12 animals, per site for the CMT, AD, CING, BARR, VIS, and EEGs; 2588 ± 11 spindles, 6 animals, for the TRN and VB). Error bars indicate mean ± S.E.M.

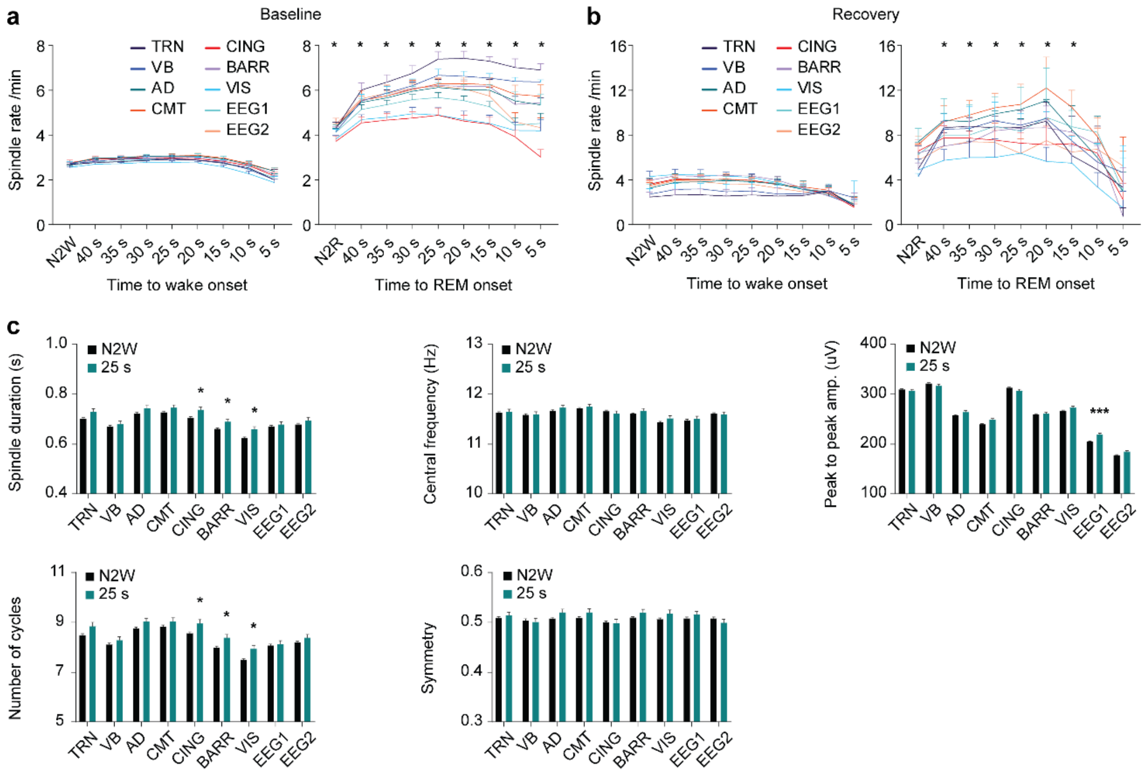

Supplementary Fig. 4. Quantification of NREM spindles before transition to wake and REM sleep. (a) Region-specific spindle rates during different time windows before transition to REM sleep (N2R) and wake (N2W), ranging from 40 s to 5 s windows before transition. Spindle rate during N2R and the entire studied time windows before REM onset is significantly higher than during N2W (5-40 s before REM onset *vs.* N2W: $P < 0.001$; $F = 531$; d.f. = 17; two-way ANOVA with Bonferroni's *post-hoc* test; $*P < 0.05$; $n = 6/12$ animals). However, transition to wakefulness did not show any significant increase in spindle rate (5-40 s before wake onset *vs.* N2W: $P > 0.05$; $F = 531$; d.f. = 17; two-way ANOVA with Bonferroni's *post-hoc* test; $*P < 0.05$; $n = 6$ animals for TRN/VB and $n = 12$ for other sites). (b) Same as (a) but for recovery sleep (5-40 s before REM onset *vs.* N2W: $P < 0.001$; 5-40 s before wake onset *vs.* N2W: $P > 0.05$; $F = 36.4$; d.f. = 17; two-way ANOVA with Bonferroni's *post-hoc* test; $n = 4$ animals). (c) Comparison between characteristics of NREM spindles during N2W and 25 s prior to REM sleep onset, including spindle duration, frequency, amplitude, number of cycles, and symmetry. Duration and number of cycles of cortical spindles within 25 s prior to REM onset are significantly higher than of N2W spindles (25 s before REM *vs.* N2W: duration: $P < 0.05$ for CING, BARR, and VIS; $F = 40.4$; d.f. = 1; number of cycles: $P < 0.05$ for CING, BARR, and VIS; $F = 39.2$; d.f. = 1; two-way ANOVA with Bonferroni's *post-hoc* test; $*P < 0.05$, $***P < 0.001$; $n = 6$ animals for TRN/VB and $n = 12$ for other sites). Error bars indicate mean ± S.E.M.

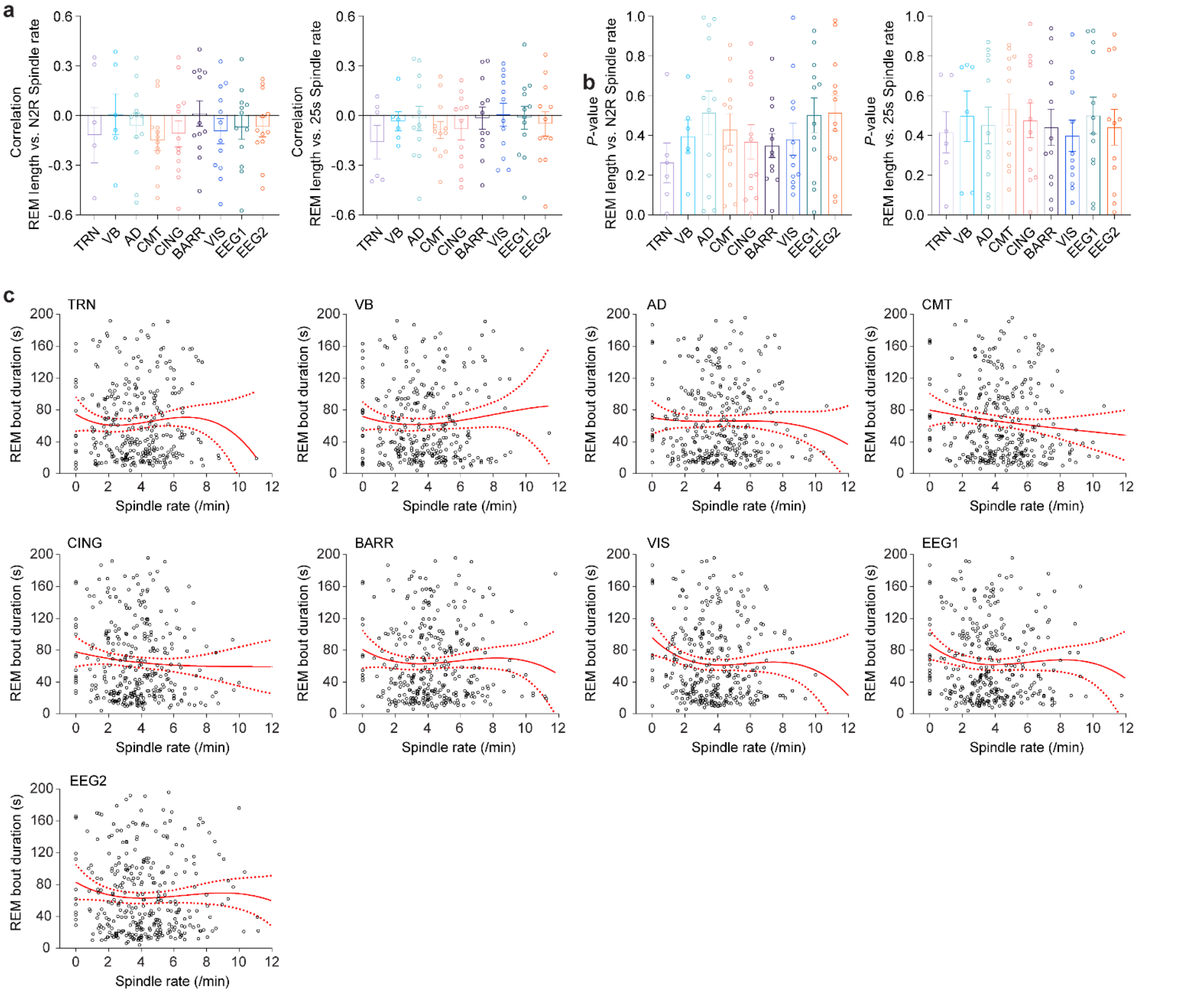

Supplementary Fig. 5. Correlation between spindle rate before REM onset and REM bout duration. (a) The bars show correlation between REM bout duration and region-specific spindle rate during N2R episodes (left) and 25 s before REM onset (right; $n = 6$ animals for TRN/VB and $n = 12$ for other sites; mean ± S.E.M.). (b) Statistical analysis shows no significant correlation between the above measures ($P > 0.05$ for all sites; mean ± S.E.M.). (c) Scatter plots show distribution of N2R spindle rate *vs.* REM bout duration, obtained from all REM episodes of all studied mice ($n = 6$ animals for TRN/VB and $n = 12$ for other sites). Each point represents one REM sleep episode and lines indicate mean ± S.E.M of points.

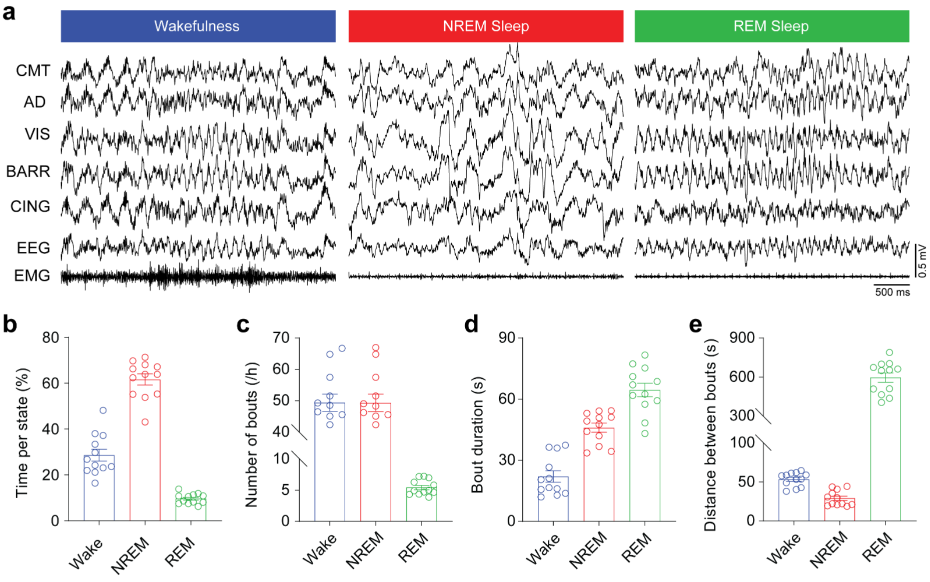

Supplementary Fig. 6. Quantification of vigilance states. (a) Representative LFP/EEG/EMG signals of wake, NREM, and REM sleep recorded from thalamic and cortical sites. (b) Percentage of time per state (wake: 28.6 ± 2.6%; NREM: 61.7 ± 2.4%; REM: 9.6 ± 0.64%; *n* = 12 animals, 5 h each). (c) Number of episodes per hour for each state (wake: 49.4 ± 2.7; NREM: 49.3 ± 2.7; REM: 5.4 ± 0.3; *n* = 12 animals, 5 h each). (d) Duration of episodes for each state (wake: 22.1 ± 2.7 s; NREM: 45.9 ± 2.2 s; REM: 64.5 ± 3.3 s; *n* = 12 animals, 5 h each). (e) Distance between episodes of each state (wake: 53.4 ± 2.5 s; NREM: 29.1 ± 2.8 s; REM: 599.2 ± 36.0 s; *n* = 12 animals, 5 h each). Bras represents mean ± S.E.M.
