# Supplementary material for: A role for spindles in the onset of rapid eye movement sleep
Source: Nat Commun. 2020 Oct 16;11:5247. doi: 10.1038/s41467-020-19076-2 (PMC7567828; doi:10.1038/s41467-020-19076-2)
Supplement: Supplementary file 3 — Description of Additional Supplementary Files [file 41467_2020_19076_MOESM3_ESM.pdf]

Description of Additional Supplementary Files

File Name: Supplementary Software
Description: Custom MATLAB script for automatic detection and characterization of spindles.
