# Supplementary material for: A role for spindles in the onset of rapid eye movement sleep
Source: Nat Commun. 2020 Oct 16;11:5247. doi: 10.1038/s41467-020-19076-2 (PMC7567828; doi:10.1038/s41467-020-19076-2)
Supplement: Supplementary file 5 — Reporting Summary [file 41467_2020_19076_MOESM5_ESM.pdf]

nature research

Corresponding author(s): Prof. Antoine Adamantidis

Last updated by author(s): Aug 31, 2020

Reporting Summary

Nature Research wishes to improve the reproducibility of the work that we publish. This form provides structure for consistency and transparency in reporting. For further information on Nature Research policies, see our Editorial Policies and the Editorial Policy Checklist.

Statistics

For all statistical analyses, confirm that the following items are present in the figure legend, table legend, main text, or Methods section.

n/a	Confirmed	
☐	☒	The exact sample size (*n*) for each experimental group/condition, given as a discrete number and unit of measurement
☐	☒	A statement on whether measurements were taken from distinct samples or whether the same sample was measured repeatedly
☐	☒	The statistical test(s) used AND whether they are one- or two-sided<br>*Only common tests should be described solely by name; describe more complex techniques in the Methods section.*
☐	☒	A description of all covariates tested
☐	☒	A description of any assumptions or corrections, such as tests of normality and adjustment for multiple comparisons
☐	☒	A full description of the statistical parameters including central tendency (e.g. means) or other basic estimates (e.g. regression coefficient) AND variation (e.g. standard deviation) or associated estimates of uncertainty (e.g. confidence intervals)
☐	☒	For null hypothesis testing, the test statistic (e.g. *F*, *t*, *r*) with confidence intervals, effect sizes, degrees of freedom and *P* value noted<br>*Give P values as exact values whenever suitable.*
☒	☐	For Bayesian analysis, information on the choice of priors and Markov chain Monte Carlo settings
☒	☐	For hierarchical and complex designs, identification of the appropriate level for tests and full reporting of outcomes
☐	☒	Estimates of effect sizes (e.g. Cohen's *d*, Pearson's *r*), indicating how they were calculated

Our web collection on statistics for biologists contains articles on many of the points above.

Software and code

Policy information about availability of computer code

Data collection	Intan RHD2000 (v 1.5.2, Intantech) and Matlab (v 2018b, Mathworks) were used for the collection of data in this study.
Data analysis	Signal processing was performed using custom written algorithms in Matlab (v. 2018b, Mathworks). Cell spiking activity was detected and sorted using the WaveClus toolbox in Matlab (https://github.com/csn-le/wave_clus). Custom Matlab scripts to perform the analysis reported in this study are available as supplementary file. Statistical analysis was performed using preset algorithms in Graphpad Prism (v. 8). Figures were prepared in Adobe Illustrator CS6.

For manuscripts utilizing custom algorithms or software that are central to the research but not yet described in published literature, software must be made available to editors and reviewers. We strongly encourage code deposition in a community repository (e.g. GitHub). See the Nature Research guidelines for submitting code & software for further information.

Data

Policy information about availability of data

All manuscripts must include a data availability statement. This statement should provide the following information, where applicable:

- Accession codes, unique identifiers, or web links for publicly available datasets
- A list of figures that have associated raw data
- A description of any restrictions on data availability

The data that support this study is available from the corresponding author upon reasonable request. The DREAMS sleep spindle database is publically available online at https://zenodo.org/record/2650142#.X0k6Icgzbg4.

Field-specific reporting

Please select the one below that is the best fit for your research. If you are not sure, read the appropriate sections before making your selection.

[x] Life sciences [ ] Behavioural & social sciences [ ] Ecological, evolutionary & environmental sciences

For a reference copy of the document with all sections, see nature.com/documents/nr-reporting-summary-flat.pdf

Life sciences study design

All studies must disclose on these points even when the disclosure is negative.

Sample size	No power calculations were performed to determine sample sizes, however similar sized cohorts were used in other relevant investigations (Herrera et al., 2016. Nat. Neuroscience, Gent et al., 2018. Nat. Neuroscience).
Data exclusions	No animals were excluded from analysis.
Replication	Different cohorts of animals per experimental condition were used to confirm replicability of results. Replication of the neuronal firing and LFPs during spindles were independent of animals during different recording sessions, and using 4 different cohorts of animals. For optogenetic experiments, 3 different cohorts of animals were used. Anatomical studies (Supplementary Fig. 1) were replicated in 5 animals, and in our previous study (Gent et al., 2018. Nat. Neuroscience). All the attempts at replication were successful.
Randomization	Animals used for LFP/unit data collection were randomly assigned to be implanted either in CMT-AD-CING-BARR-VIS or TRN-VB-BARR coordinates with multisite simultaneous optrode. Animals used for optogenetics experiments were randomly assigned to be injected/ stimulated as YFP control, TRN or AD.
Blinding	Sleep scoring using EEG and EMG signals was performed blinded to the experimental conditions. Blinding was not relevant for data collection and analysis, as each experimental condition has different recording sites.

Reporting for specific materials, systems and methods

We require information from authors about some types of materials, experimental systems and methods used in many studies. Here, indicate whether each material, system or method listed is relevant to your study. If you are not sure if a list item applies to your research, read the appropriate section before selecting a response.

Materials & experimental systems

n/a	Involved in the study	
[ ]	[x]	Antibodies
[x]	[ ]	Eukaryotic cell lines
[x]	[ ]	Palaeontology and archaeology
[ ]	[x]	Animals and other organisms
[x]	[ ]	Human research participants
[x]	[ ]	Clinical data
[x]	[ ]	Dual use research of concern

Methods

n/a	Involved in the study	
[x]	[ ]	ChIP-seq
[x]	[ ]	Flow cytometry
[x]	[ ]	MRI-based neuroimaging

Antibodies

Antibodies used	The following commercially available antibodies were used:<br>Primary: Chicken IgY-anti GFP (Invitrogen A10262, lot no. 45351A), dilution 1:4000<br>Secondary: Goat-anti Chicken IgY 488 (Abcam ab96947, lot no. GR263078-11), dilution 1:500
Validation	Concentrations were used based on validation in mice in previous publications (see: Jego et al., 2013 Nat. Neuroscience; Herrera et al., 2016 Nat. Neuroscience, Gent et al., 2018 Nat. Neuroscience).

Animals and other organisms

Policy information about studies involving animals; ARRIVE guidelines recommended for reporting animal research

Laboratory animals	Adult (> 6weeks) male C57BL6 and Tg(VGAT)::IRES-Cre mice.
Wild animals	No wild animals were used in this study.
Field-collected samples	No field samples were collected for analysis in this study.

Ethics oversight	Animals were treated according to protocols and guidelines approved by the Veterinary office of the Canton of Bern, Switzerland (License number BE 49/7).

Note that full information on the approval of the study protocol must also be provided in the manuscript.
